# Characteristics of insufficiently active participants that benefit from health-enhancing physical activity (HEPA) promotion programs implemented in the sports club setting

**DOI:** 10.1186/s12889-018-5579-2

**Published:** 2018-06-01

**Authors:** Linda Ooms, Chantal Leemrijse, Dorine Collard, Nicolette Schipper-van Veldhoven, Cindy Veenhof

**Affiliations:** 1 Netherlands Institute for Health Services Research (NIVEL), PO Box 1568, 3500 BN Utrecht, the Netherlands; 2 Mulier Institute, PO Box 85445, 3508 AK Utrecht, the Netherlands; 3 Windesheim University, Research Group for Sport Pedagogy, PO Box 10090, 8000 GB Zwolle, the Netherlands; 4 Netherlands Olympic Committee and Netherlands Sports Federation (NOC*NSF), PO Box 302, 6800 AH Arnhem, the Netherlands; 5 Physical Therapy Research, Department of Rehabilitation, Physiotherapy Sciences & Sports, University Medical Center Utrecht, Utrecht University, PO Box 85500, 3508 GA Utrecht, the Netherlands; 6 Expertise Center Innovation of Care, Research Group Innovation of Mobility Care, University of Applied Sciences Utrecht, PO Box 12011, 3501 AA Utrecht, the Netherlands

**Keywords:** Sports club, Sporting program, Health-enhancing physical activity, Insufficiently active

## Abstract

**Background:**

Health-enhancing physical activity (HEPA) promotion programs are implemented in sports clubs. The purpose of this study was to examine the characteristics of the insufficiently active participants that benefit from these programs.

**Methods:**

Data of three sporting programs, developed for insufficiently active adults, were used for this study. These sporting programs were implemented in different sports clubs in the Netherlands. Participants completed an online questionnaire at baseline and after six months (*n* = 458). Of this sample, 35.1% (*n* = 161) was insufficiently active (i.e. not meeting HEPA levels) at baseline. Accordingly, two groups were compared: participants who were insufficiently active at baseline, but increased their physical activity to HEPA levels after six months (activated group, *n* = 86) versus participants who were insufficiently active both at baseline and after six months (non-activated group, *n* = 75). Potential associated characteristics (demographic, social, sport history, physical activity) were included as independent variables in bivariate and multivariate logistic regression analyses.

**Results:**

The percentage of active participants increased significantly from baseline to six months (from 64.9 to 76.9%, *p* < 0.05). The bivariate logistic regression analyses showed that participants in the activated group were more likely to receive support from family members with regard to their sport participation (62.8% vs. 42.7%, *p* = 0.02) and spent more time in moderate-intensity physical activity (128 ± 191 min/week vs. 70 ± 106 min/week, *p* = 0.02) at baseline compared with participants in the non-activated group. These results were confirmed in the multivariate logistic regression analyses: when receiving support from most family members, there is a 216% increase in the odds of being in the activated group (OR = 2.155; 95% CI: 1.118–4.154, *p* = 0.02) and for each additional 1 min/week spent in moderate-intensity physical activity, the odds increases with 0.3% (OR = 1.003; 95% CI: 1.001–1.006, *p* = 0.02).

**Conclusions:**

The results suggest that HEPA sporting programs can be used to increase HEPA levels of insufficiently active people, but it seems a challenge to reach the least active ones. It is important that promotional strategies and channels are tailored to the target group. Furthermore, strategies that promote family support may enhance the impact of the programs.

## Background

Globally, insufficient physical activity is a major risk factor for mortality and non-communicable diseases, such as cardiovascular diseases, cancer and diabetes [1]. According to international physical activity guidelines, adults should do at least 150 min of moderate-intensity aerobic physical activity throughout the week or do at least 75 min of vigorous-intensity aerobic physical activity throughout the week [2]. The guidelines can also be met by a comparable amount of both moderate- and vigorous-intensity physical activity. However, research indicates that a third of the world’s population is not meeting these levels of health-enhancing physical activity (HEPA) [3]. Considering the importance of regular physical activity in the prevention of mortality and non-communicable diseases, these findings are alarming [1, 4, 5].

Consequently, HEPA promotion is a priority aim of the World Health Organization (WHO), other health professionals and policy makers in different countries [1, 6–10]. According to the WHO, health should be promoted in the places where people live, learn, work and play [11]. Therefore, HEPA should also be stimulated in different settings. The involvement of the organized sports sector, and in particular the sports club, as a setting for HEPA promotion is a new strategy implemented by health professionals and policy makers [6, 7, 9, 10]. Due to their wide reach, their informal learning environment and the voluntary nature of participation, sports clubs have great potential in promoting HEPA in the population [12, 13]. Nonetheless, sport participation is characterized by considerable inequalities. Participation rates are lower among women, decline with age and are reduced in people with chronic diseases, low levels of education and people from culturally diverse backgrounds [14–16]. Concurrently, the people in these population subgroups are also more likely to be insufficiently active and at higher risk for developing non-communicable diseases [1, 3, 4, 17]. Thus, increasing these target groups’ HEPA levels through participation in sport at a sports club may be challenging.

In the research literature, some examples of HEPA promotion strategies in the sports club setting can be found [6, 7, 10, 18]. There is some evidence that relatively short sporting programs, implemented by sports clubs, can be used to encourage insufficiently active people to engage in and continue sport at HEPA levels [19, 20]. However, these studies considered the participant population as a whole. Therefore, the purpose of this study was to examine the characteristics of the insufficiently active participants that benefit from these programs in terms of increasing HEPA. The study results can contribute to developing effective and tailored sporting programs aimed at insufficiently active people. In addition, the findings will guide health professionals, policy makers and sport practitioners in their choices for HEPA promotion strategies regarding this target group.

## Methods

### Study population

**Fig. 1:**
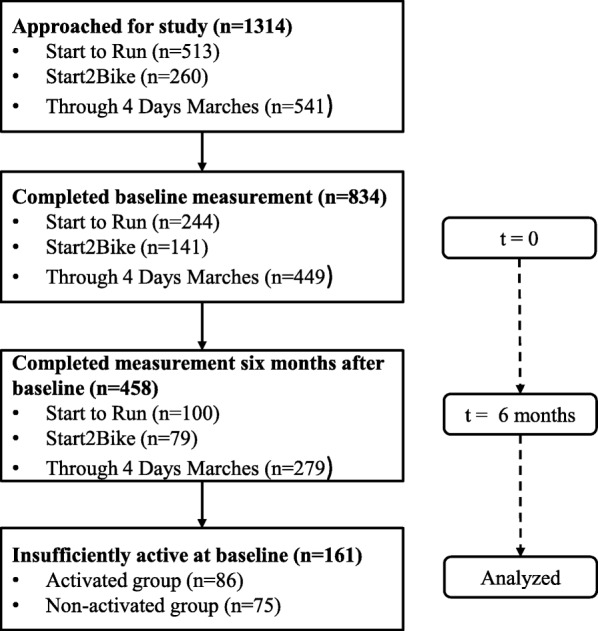
Participant flow through the study. Active: participants who met the Combi-norm; Insufficiently active: participants who did not meet the Combi-norm; Activated group: participants who were insufficiently active at baseline, but active after six months; Non-activated group: participants who were insufficiently active both at baseline and after six months

Data from participants of three sporting programs, aimed at insufficiently active adults, were used, namely Start to Run, Start2Bike and Through 4 Days Marches (see Table 1). These data were collected in light of a larger study in which both a process and effectiveness evaluation of the programs were conducted [10, 19, 20]. In this study, more in-depth analyses of the data were performed. The data of the individual sporting programs were combined into one dataset to increase statistical power of the study (i.e. the number of insufficiently active people per sporting program was relatively low). The datasets could be combined because the programs were all adapted to insufficiently active adults, using feasible sports (i.e. running, sportive cycling and walking can be done anywhere and at any time), similar training principles and similar strategies to retain participants. Dutch National Sports Federations (NSFs) started the programs within the National Action Plan for Sport and Exercise which was aimed at increasing the number of people meeting HEPA levels [10]. Different sports clubs implemented the programs in the period 2008–2011. Online questionnaires were sent to participants at the start of the programs (spring 2009) and after six months. The NSFs provided e-mail addresses of participants who had subscribed for the programs in spring 2009 (*n* = 1314). The baseline questionnaire contained detailed information about the background and aims of the study. In addition, participants were informed that participation was voluntary, all collected information would be kept strictly confidential and only anonymized data would be published. In case of questions about the research, they could contact the researcher by email or telephone. By completing the baseline questionnaire, participants gave consent for participation in the study (*n* = 834). In total, 458 participants finished both questionnaires and formed the initial sample of this study (see Fig. 1 for the flow of participants through the study). Non-response analyses showed that program participants who did not complete the six-month measurement were more likely to be female (71.0% female vs. 57.0% female) and significantly younger (41 ± 11 years vs. 45 ± 11 years) compared with participants that did complete this measurement. Furthermore, demographic data collected by the NSFs confirmed that the participants of this study were representative for the entire participant population of the individual sporting programs with regard to age and sex.

**Table 1:** Description of sporting programs

National Sports Federation	Sporting program	Description	Frequency and duration of activities	Intensity of activities
Athletics	Start to Run	Six-week training program for inactive adults and adult novice runners. The program is aimed at learning the basic skills of running and gradually increasing (aerobic) fitness of participants. At the end of the program, participants should be able to run 3 km continuously and they are offered the possibility to test their running abilities in a 3 km test run. The program is offered two times a year (in March and September) by athletics clubs.	▪ Three times a week of running: one time under guidance of a professional coach, two times individually. Training sessions last for 1,5 h (guided sessions) and 45 min (individual sessions). ▪ A guided session consists of 30 min of theory, followed by 1 h of practice. ▪ Emails with training instructions and theory are sent to the participants on a weekly basis.	VPA (i.e. jogging/running) and MPA (i.e. walking) are alternated. During the training period, the frequency of the VPA bouts gradually decrease, but their duration and intensity (from jogging to running) increase. For example: Week 1, guided training: 8 × 1 min jogging, with 3 min walking rest breaks. Week 5, guided training: 3 × 10 min jogging/running, with 5 min walking rest breaks.
Sportive cycling	Start2Bike	Six-week training program for inactive adults and adult novice cyclers (road cycling, mountain biking). The program is aimed at learning the basic skills of road cycling or mountain biking and gradually increasing (aerobic) fitness of participants. At the end of the program, participants can participate in a cycling event. The program is offered two times a year (in March and September) by (sportive) cycling clubs.	▪ Three times a week of cycling: one time under guidance of a professional coach, two times individually. Training sessions last for 2 h. ▪ A guided session consists of theory and practice. Theory items are discussed during practice. ▪ Emails with training instructions and theory are sent to the participants on a weekly basis.	VPA (i.e. road cycling or mountain biking at a high speed) and MPA (i.e. road cycling or mountain biking at a low speed; practicing cycling skills) are alternated. During the training period, the duration and intensity of the VPA bouts are gradually increased. For example: Week 1, guided training mountain biking: learning to break and how to use your gears; cycling 10 km at a comfortable speed. Week 5, guided training mountain biking: learning to climb and descend; cycling 25 km at a higher speed.
Walking	Through 4 Days Marches	Six-month training program for (inactive) adults willing to participate in the Four Days Marches of Nijmegen (four consecutive days of walking with 30 km, 40 km and 50 km walking distances). During the training period, participants can take part in two walking events to test their walking abilities. Participants can take part in the program individually or at a walking club. The program is offered one time a year.	▪ Two to five times a week of walking for 1–5 h depending on walking distance at the event and period of training program. ▪ Theory items are discussed during guided sessions and test events. ▪ Training instructions and theory are displayed on a website, which is available to all participants at any time.	MPA (i.e. walking). During the training period, the frequency, duration and intensity of the MPA bouts are gradually increased. For example, training for 40 km walking distance: Week 1: 1 h of walking two times a week at a low walking speed. Week 7: 1,5 h of walking three times a week at a higher walking speed.

*MPA* Moderate-intensity physical activity, *VPA* Vigorous-intensity physical activity

This study followed ethical principles (i.e. with regard to enabling participation, informed consent, confidentiality, avoiding undue intrusion, avoiding adverse consequences and data protection) [21]. Participants were not exposed to procedures, nor were they obligated to follow certain behavioral rules (i.e. participants were approached for the study after they had voluntarily registered for participation in the sporting programs). Therefore, in accordance with Dutch law, medical ethics committee’s approval was not mandatory for conducting this study [22]. Study privacy procedures followed Dutch Data Protection Authority regulations. The Transparent Reporting of Evaluations with Nonrandomized Designs (TREND) group reporting standards were used as guidance for reporting of results [23].

### Sporting programs

A description of the sporting programs can be found in Table 1. The three sporting programs were developed for insufficiently active adults. The threshold for participation was very low so that adults with no previous training experiences or specific sport skills could participate. In this regard, graded training programs were applied, starting with (very) small amounts of moderate- and/or vigorous-intensity physical activity and gradually increasing intensity and duration of physical activity over time. The physical activities were always adapted to the (physical) abilities of the participants. At the sports club level, sometimes multiple groups were formed with each group providing to a different level of beginner. At the end of the programs, participants could test their sporting abilities by participating in a (test) sporting event (e.g. a 3 km test run). All programs consisted of practice and theory. The practical part consisted of a warming-up, practicing of specific sport skills and cooling-down. There was one group training session each week guided by one or more professional coaches, two or more training sessions were performed individually. Participants received instructions for the individual training sessions from their coaches (face-to-face and through e-mail). Theory items, such as the health benefits of sport participation, healthy food and drinks and prevention of injuries, were discussed with the group before, during or after practice. At the final training session, participants received information about club membership and were encouraged by their coaches (both verbally and through email) to continue participating in the particular sport. Continuation was possible at the club (at reduced costs) in an appropriate beginners’ group. Participation in the programs was at low costs and, for the Start2Bike program, some sports clubs provided cycles to participants during the training program. The NSFs provided trainer courses, especially for the sporting programs, to educate trainers about how to guide the insufficiently active target group. At a national level, the programs were promoted to (potential) participants via national press, websites of the NSFs and social media. Sports clubs recruited participants using different recruitment strategies, like the placement of advertisements in local media and the distribution of posters and flyers in the neighborhood.

### Possible associated characteristics

The following characteristics were measured in the baseline questionnaire and included in analyses: *demographic characteristics*, *sport history* and *physical activity. Social factors* were also measured in the baseline questionnaire. Since *social factors*, like social support from family and friends, are related to physical activity behavior in a number of studies [24–28], these factors were also included in the analyses. The characteristics will be described in more detail below.

### Demographic characteristics

Participants were asked to report their sex, age, educational attainment (highest level completed) and the presence of chronic diseases (yes/no). Education was categorized into low-average (high school or lower) and high (higher professional education and university). Due to the low number of participants with low education levels, it was not possible to present this as a separate category in the analysis. Body Mass Index (BMI), defined as weight (kg) divided by height squared (m^2^), was calculated from participants’ self-reported height and weight. According to WHO standards, participants with a BMI ≥ 25 were classified as overweight [29]. Ethnicity was determined by country of birth of participant’s parents. Based on the standard definition of ethnicity of Statistics Netherlands [30], participants were divided in people with a Dutch background (i.e. both parents are born in the Netherlands) and people with a foreign background (i.e. at least one parent is born abroad).

### Social factors

Social factors consisted of frequency of sport participation of *most* family members and friends. This was questioned as follows: *Most* of my family members/friends participate in sport: ‘never’, ‘one time a week’, ‘a few times a week’ or ‘every day’. Sport participation was defined as regularly if *most* members of the group participated in sport at least once a week. In addition, it was questioned whether *most* family members/friends supported the respondent participating in sport: *Most* of my family members/friends support me participating in sport: ‘yes’ or ‘no’.

### Sport history

Participants’ sport history was assessed by measuring sport participation (yes/no) and membership of a sports club (yes/no) before involvement in the program.

### Physical activity

The *S*hort *QU*estionnaire to *AS*sess *H*ealth-enhancing physical activity (SQUASH) was used to measure physical activity levels of participants at baseline and after six months. This questionnaire is seen as sufficiently reliable and valid to measure adults’ physical activity levels [31]. More details of the SQUASH-procedure can be found in a previous research article [19]. In short, the SQUASH measures physical activity levels for a regular week in the past month. It includes five physical activity domains (commuting activities, leisure-time activities, sport activities, household activities and activities at work and school) and three main queries (days per week, average time per day, self-reported intensity: light, moderate, vigorous). Accordingly, total minutes of physical activity per week and the minutes per week spent in each intensity category were calculated. To measure HEPA levels, the Dutch physical activity norms were used [17]. These norms are based on the international physical activity guidelines and recommend that adults should undertake a minimum of 30 min of moderate-intensity physical activity on five days per week (Dutch Norm for Health-enhancing Physical Activity: DNHPA) or 20 min of vigorous-intensity physical activity on three days per week (Fit-norm) for health benefits. Someone who meets at least one of the two norms adheres to the so-called ‘Combi-norm’, the third norm used in the Netherlands (see also Table 2) [2, 17]. A participant was classified as ‘active’ when he or she met the Combi-norm, because this norm combines both the DNHPA and Fit-norm and indicates whether someone is sufficiently active. Participants not meeting this norm were categorized as ‘insufficiently active’.

**Table 2:** Dutch physical activity norms for adults (≥ 18 years)

Norm	Description
Dutch Norm for Health-enhancing Physical Activity (DNHPA)	*Adults (18–54 years):* Thirty minutes or more of at least moderate-intensity aerobic (endurance) physical activity (≥ 4 MET) on at least five days each week. *Adults (55 years and older):* Thirty minutes or more of at least moderate-intensity aerobic (endurance) physical activity (≥ 3 MET) on at least five days each week. A moderate-intensity aerobic physical activity requires a moderate amount of effort and noticeably accelerates the heart rate, e.g. brisk walking, gardening.
Fit-norm	*Adults (18–54 years):* Twenty minutes or more of vigorous-intensity physical activity (≥ 6.5 MET) on at least three days each week. *Adults (55 years and older):* Twenty minutes or more of vigorous-intensity physical activity (≥ 5 MET) on at least three days each week. A vigorous-intensity physical activity requires a large amount of effort and causes rapid breathing and a substantial increase in heart rate, e.g. running, mountain biking and road cycling.
Combi-norm	Meeting the DNHPA and/or Fit-norm. An adult is physically active enough to improve and maintain health when he or she meets at least one of the above mentioned norms (i.e. the DNHPA or Fit-norm).

*MET* Metabolic equivalent

### Statistical analyses

Stata statistical software (version 10.1, Stata Corporation, College Station, Texas) was used for statistical analyses. The main characteristics of study participants were described using descriptive statistics. Of the 458 participants included in this study, 35.1% (*n* = 161) were insufficiently active at baseline. These participants were selected to examine baseline characteristics associated with meeting HEPA levels after six months. For this purpose, two groups were compared: participants who were insufficiently active at baseline, but active after six months (activated group) versus participants who were insufficiently active both at baseline and after six months (non-activated group). First, bivariate logistic regression analyses were used with potential associated characteristics as independent variables and group (activated group vs. non-activated group, with the latter as reference category) as dependent variable. Subsequently, all variables with a *P*-value < 0.15 were entered into a multivariate model, after which backwards elimination of variables was performed, removing the variable with the least significant *P*-value (P to remove ≥ 0.10). This was done until only variables with a *P*-value < 0.05 remained. The type of sporting program (Start to Run, Start2Bike, Through 4 Days Marches) was included as independent variable in all logistic regression analyses to control for possible differences between sporting programs. Dummy variables were created for this purpose, with the Start to Run program serving as reference category. Statistical significance was set at a *P*-value < 0.05.

## Results

### Baseline characteristics of participants and changes in physical activity

Baseline characteristics of the participants are presented in Table 3. Of the 458 participants included in this study, 35.1% (*n* = 161) were insufficiently active. The percentage of active participants increased significantly from baseline to six months (from 64.9 to 76.9%, *p* < 0.05) (see also Table 3).

**Table 3:** Baseline characteristics of participants and changes in physical activity

	All sporting programs combined	Start to Run	Start2Bike	Through 4 Days Marches
Sample size (n)	458	100	79	279
Demographic characteristics				
Gender n, (%)				
Female	261 (57.0)	70 (70.0)	26 (32.9)	165 (59.1)
Male	197 (43.0)	30 (30.0)	53 (67.1)	114 (40.9)
Age, mean ± SD (years)	45 ± 11	40 ± 10	45 ± 9	46 ± 11
Overweight (BMI ≥ 25) n, (%)^a^				
Yes	201 (44.0)	40 (40.4)	33 (41.8)	128 (45.9)
No	256 (56.0)	59 (59.6)	46 (58.2)	151 (54.1)
Ethnicity n, (%)^b^				
Dutch	357 (93.7)	70 (88.6)	68 (95.8)	219 (94.8)
Foreign	24 (6.3)	9 (11.4)	3 (4.2)	12 (5.2)
Chronic diseases n, (%)^b^				
Yes	52 (13.6)	9 (11.4)	9 (12.5)	34 (14.7)
No	331 (86.4)	70 (88.6)	63 (87.5)	198 (85.3)
Education n, (%)^b^				
Low-average	224 (58.6)	42 (53.2)	36 (50.0)	146 (63.2)
High	158 (41.4)	37 (46.8)	36 (50.0)	85 (36.8)
Social factors				
Regular sports participation of most… n, (%)				
Family members				
Yes	335 (73.1)	76 (76.0)	53 (67.1)	206 (73.8)
No	123 (26.9)	24 (24.0)	26 (32.9)	73 (26.2)
Friends				
Yes	354 (77.3)	78 (78.0)	54 (68.4)	222 (79.6)
No	104 (22.7)	22 (22.0)	25 (31.7)	57 (20.4)
Supporting participant’s sport participation by most… n, (%)				
Family members				
Yes	236 (51.5)	58 (58.0)	42 (53.2)	136 (48.8)
No	222 (48.5)	42 (42.0)	37 (46.8)	143 (51.3)
Friends				
Yes	238 (52.0)	52 (52.0)	38 (48.1)	148 (53.1)
No	220 (48.0)	48 (48.0)	41 (51.9)	131 (47.0)
Sport history				
Participation in sport before program n, (%)				
Yes	325 (71.0)	55 (55.0)	53 (67.1)	217 (77.8)
No	133 (29.0)	45 (45.0)	26 (32.9)	62 (22.2)
Member of a sports club n, (%)				
Yes	229 (50.0)	47 (47.0)	37 (46.8)	145 (52.0)
No	229 (50.0)	53 (53.0)	42 (53.2)	134 (48.0)
Physical activity				
Light-intensity PA, mean ± SD (min/week)				
Baseline	1939 ± 1348	1814 ± 1224	1940 ± 1313	1983 ± 1402
After six months	1950 ± 1297	1947 ± 1043	1760 ± 1350	2004 ± 1362
Moderate-intensity PA, mean ± SD (min/week)				
Baseline	370 ± 615	213 ± 453	329 ± 586	438 ± 662
After six months	445 ± 638*	206 ± 369	300 ± 507	571 ± 712*
Vigorous-intensity PA, mean ± SD (min/week)				
Baseline	275 ± 320	238 ± 250	358 ± 368	264 ± 325
After six months	340 ± 345*	382 ± 306*	475 ± 405*	286 ± 328
Total time spent in PA, mean ± SD (min/week)				
Baseline	2583 ± 1432	2265 ± 1251	2626 ± 1372	2685 ± 1497
After six months	2734 ± 1400*	2536 ± 1210*	2535 ± 1372	2862 ± 1459*
Active n, (%)				
Baseline	297 (64.9)	58 (58.0)	55 (69.6)	184 (66.0)
After six months	352 (76.9)*	84 (84.0)*	64 (81.0)*	204 (73.1)*

*BMI* Body Mass Index, *PA* Physical activity, *SD* Standard deviation

^a^ BMI could not be calculated for one respondent

^b^ n < sample n, because questions were not mandatory. Consequently, not all participants completed these questions

* Significant (*p* < 0.05) difference: after six months vs. baseline

### Baseline characteristics of insufficiently active participants in comparison with active participants

Baseline characteristics of insufficiently active participants in comparison with active participants are presented in Table 4. There were significant differences between these two groups: insufficiently active participants were more likely to be overweight (50.9% vs. 40.2%, *p* = 0.02), had less friends that participated in sport regularly (71.4% vs. 80.5%, *p* = 0.02) and were less likely to be participating in sport before involvement in the sporting program (54.0% vs. 80.1%, *p* < 0.001). In addition, they spent less of their time in physical activity (2128 ± 1378 min/week vs. 2829 ± 1403 min/week, *p* < 0.001), in this case moderate- (101 ± 159 min/week vs. 516 ± 714 min/week, *p* < 0.001) and vigorous-intensity (60 ± 79 min/week vs. 391 ± 341 min/week, *p* < 0.001) physical activity.

**Table 4:** Baseline characteristics: insufficiently active participants vs. active participants

	Insufficiently active participants	Active participants	*P*-value^a^
Sample size (n)	161	297	
Demographic characteristics			
Gender n, (%)			
Female	85 (52.8)	176 (59.3)	0.08
Male	76 (47.2)	121 (40.7)	
Age, mean ± SD (years)	43 ± 10	46 ± 12	0.08
Overweight (BMI ≥ 25) n, (%)^b^			
Yes	82 (50.9)	119 (40.2)	0.02*
No	79 (49.1)	177 (59.8)	
Ethnicity n, (%)^c^			
Dutch	133 (93.0)	224 (94.1)	0.79
Foreign	10 (7.0)	14 (5.9)	
Chronic diseases n, (%)^c^			
Yes	23 (16.0)	29 (12.1)	0.28
No	121 (84.0)	210 (87.9)	
Education n, (%)^c^			
Low-average	77 (53.9)	147 (61.5)	0.14
High	66 (46.2)	92 (38.5)	
Social factors			
Regular sports participation of most… n, (%)			
Family members			
Yes	111 (68.9)	224 (75.4)	0.11
No	50 (31.1)	73 (24.6)	
Friends			
Yes	115 (71.4)	239 (80.5)	0.02*
No	46 (28.6)	58 (19.5)	
Supporting participant’s sport participation by most… n, (%)			
Family members			
Yes	86 (53.4)	150 (50.5)	0.61
No	75 (46.6)	147 (49.5)	
Friends			
Yes	92 (57.1)	146 (49.2)	0.11
No	69 (42.9)	151 (50.8)	
Sport history			
Participation in sport before program n, (%)			
Yes	87 (54.0)	238 (80.1)	< 0.001*
No	74 (46.0)	59 (19.9)	
Member of a sports club n, (%)			
Yes	71 (44.1)	158 (53.2)	0.07
No	90 (55.9)	139 (46.8)	
Physical activity			
Light-intensity PA, mean ± SD (min/week)	1967 ± 1361	1923 ± 1343	0.68
Moderate-intensity PA, mean ± SD (min/week)	101 ± 159	516 ± 714	< 0.001*
Vigorous-intensity PA, mean ± SD (min/week)	60 ± 79	391 ± 341	< 0.001*
Total time spent in PA, mean ± SD (min/week)	2128 ± 1378	2829 ± 1403	< 0.001*

*BMI* Body Mass Index, *PA* Physical activity, *SD* Standard deviation

^a^*P*-value adjusted for sporting program

^b^ BMI could not be calculated for one respondent

^c^ n < sample n, because questions were not mandatory. Consequently, not all participants completed these questions

* Significant (*p* < 0.05) difference between insufficiently active participants and active participants

### Comparison of activated group vs. non-activated group: results bivariate logistic regression analyses

Table 5 compares the activated group (participants who were insufficiently active at baseline, but active after six months) with the non-activated group (participants who were insufficiently active both at baseline and after six months) on baseline characteristics. Based on the bivariate logistic regression analyses, significant differences between these groups were found: participants in the activated group were more likely to receive support from family members with regard to their sport participation (62.8% vs. 42.7%, *p* = 0.02) and spent more time in moderate-intensity physical activity (128 ± 191 min/week vs. 70 ± 106 min/week, *p* = 0.02) at baseline compared with participants in the non-activated group.

**Table 5:** Baseline characteristics: activated group vs. non-activated group, results bivariate logistic regression analyses

	Activated group	Non-activated group	*P*-value^a^
Sample size (n)	86	75	
Demographic characteristics			
Gender n, (%)			
Female	43 (50.0)	42 (56.0)	0.40
Male	43 (50.0)	33 (44.0)	
Age, mean ± SD (years)	44 ± 10	43 ± 9	0.42
Overweight (BMI ≥ 25) n, (%)^b^			
Yes	47 (54.7)	35 (46.7)	0.30
No	39 (45.4)	40 (53.3)	
Ethnicity n, (%)^c^			
Dutch	70 (94.6)	63 (91.3)	0.32
Foreign	4 (5.4)	6 (8.7)	
Chronic diseases n, (%)^c^			
Yes	12 (16.2)	11 (15.7)	0.85
No	62 (83.8)	59 (84.3)	
Education n, (%)^c^			
Low-average	41 (56.2)	36 (51.4)	0.47
High	32 (43.8)	34 (48.6)	
Social factors			
Regular sports participation of most… n, (%)			
Family members			
Yes	55 (64.0)	56 (74.7)	0.14
No	31 (36.1)	19 (25.3)	
Friends			
Yes	59 (68.6)	56 (74.7)	0.43
No	27 (31.4)	19 (25.3)	
Supporting participant’s sport participation by most… n, (%)			
Family members			
Yes	54 (62.8)	32 (42.7)	0.02*
No	32 (37.2)	43 (57.3)	
Friends			
Yes	55 (64.0)	37 (49.3)	0.07
No	31 (36.1)	38 (50.7)	
Sport history			
Participation in sport before program n, (%)			
Yes	45 (52.3)	42 (56.0)	0.83
No	41 (47.7)	33 (44.0)	
Member of a sports club n, (%)			
Yes	40 (46.5)	31 (41.3)	0.49
No	46 (53.5)	44 (58.7)	
Physical activity			
Light-intensity PA, mean ± SD (min/week)	1940 ± 1601	1998 ± 1030	1.0
Moderate-intensity PA, mean ± SD (min/week)	128 ± 191	70 ± 106	0.02*
Vigorous-intensity PA, mean ± SD (min/week)	68 ± 87	51 ± 68	0.14
Total time spent in PA, mean ± SD (min/week)	2136 ± 1610	2119 ± 1061	0.70

*BMI* Body Mass Index, *PA* Physical activity, *SD* Standard deviation

^a^*P*-value for difference between activated group and non-activated group based on bivariate logistic regression analyses. The potential associated characteristic was included as independent variable and group (activated group vs. non-activated group, with the latter as reference category) as dependent variable. Corrections were made for type of sporting program

^b^ BMI could not be calculated for one respondent

^c^ n < sample n, because questions were not mandatory. Consequently, not all participants completed these questions

* Significant (*p* < 0.05) difference between activated group and non-activated group

### Comparison of activated group vs. non-activated group: results multivariate (backwards) logistic regression analyses

Table 6 presents the results of the multivariate (backwards) logistic regression analyses. These analyses confirmed the bivariate logistic regression analyses: participants in the activated group were more likely to receive support from family members with regard to their sport participation and spent on average more minutes in moderate-intensity physical activity at baseline compared with participants in the non-activated group. More specifically, when receiving support from most family members there is a 216% increase in the odds of being in the activated group (OR = 2.155; 95% CI: 1.118–4.154, *p* = 0.02) and for each additional 1 min/week spent in moderate-intensity physical activity, the odds increases with 0.3% (OR = 1.003; 95% CI: 1.001–1.006, *p* = 0.02) (see Table 6).

**Table 6:** Baseline characteristics: activated group vs. non-activated group, results multivariate (backwards) logistic regression analyses

	OR (95% CI)^a^	*P*-value^b^
Social factors		
Supporting participant’s sport participation by most…		
Family members	2.155 (1.118–4.154)	0.02*
Physical activity		
Moderate-intensity PA	1.003 (1.001–1.006)	0.02*

*CI* Confidence interval, *OR* Odds ratio, *PA* Physical activity

^a^OR activated group vs. non-activated group, with the latter group as the reference category

^b^*P*-value for difference between activated group and non-activated group based on multivariate (backwards) logistic regression analyses. The potential associated characteristics were included as independent variables, using backwards elimination of variables, and group (activated group vs. non-activated group, with the latter as reference category) as dependent variable. Corrections were made for type of sporting program

*Significant (*p* < 0.05) difference between activated group and non-activated group

## Discussion

### General findings

To our knowledge, this is the first study examining the characteristics of the insufficiently active participants that benefit from HEPA promotion programs implemented in the sports club setting. Results showed that a third of the participants was insufficiently active at baseline. The percentage of participants meeting HEPA levels increased significantly during the six-month study period. Insufficiently active participants were more likely to meet HEPA levels after six months when they received support from family members with regard to their sport participation and spent more time in moderate-intensity physical activity at baseline.

### Explanation of findings and practical implications

Social support from family to be physically active has been associated with regular physical activity participation and initiation of this behavior [24–28]. For instance, family members can influence sport behavior positively by providing social norms that enable this behavior or by providing positive feedback about (the benefits of) the participant’s sport participation [26]. Therefore, promoting social support from family as a component in HEPA strategies may be advantageous. The examined sporting programs did not use particular social support strategies. Nonetheless, the sports club itself can be an ideal setting to involve other family members as passive or active participants. Introducing family members to the activity at an introductory session, using them as (sporting) buddies or involving them in other club activities (social activities, volunteering), may create the necessary support for the insufficiently active participant to participate in sport. Indeed, sports club activities can include multiple family members [12]. However, from the literature it is not known how much and what kind of support is necessary to initiate sport behavior [25]. Furthermore, the relationship between physical activity and social support is a dynamic process in which the amount and type of support may change over time and through the phases of adoption and maintenance of this behavior [24, 32]. Therefore, research should examine which family support strategies at which stages of behavioral change are most beneficial to increase participant’s sport behavior.

The results suggest that the sporting programs can be used to increase HEPA levels of insufficiently active people, especially of those who already engage in a modest amount of moderate-intensity physical activity. Nonetheless, a majority of the participants was already sufficiently active at baseline and specific insufficiently active population subgroups (e.g. older adults, people with chronic diseases, people with a foreign background, lower educated people) were hardly reached. Barriers and preferences for sport and physical participation vary across different population subgroups [33]. For insufficiently active people, unfamiliarity with the sport setting or the ‘tough’ image of sport may prevent them from participating [10, 33]. Moreover, there is evidence that the target group is less aware of sport and physical activity opportunities in their neighborhood [34]. Therefore, an explanation for not reaching large numbers of insufficiently active people, might be the use of inappropriate recruitment strategies. For these sporting programs, participants were recruited by sports clubs using different recruitment strategies (e.g. the placement of advertisements in local media and the distribution of posters and flyers in the neighborhood). Not knowing the right people or channels to reach insufficiently active people was indeed a barrier for recruitment of this target group by the sports clubs [10].

To increase the population prevalence of HEPA it is important to attract more insufficiently active people to these HEPA sporting programs. Therefore, sports clubs should promote the sport activities in a non-threatening and fun manner, using promotion channels that are appropriate to the target group. In this regard, they may consider engaging in partnerships with primary health care, community health or other relevant organizations to get closer to this target group [18]. For instance, physicians and other health professionals can refer patients who need to be more physically active for their health to the sporting programs [35].

Furthermore, for people who are completely inactive, it is not inconceivable that more comprehensive strategies may be necessary to increase their physical activity levels. The threshold for sport participation can be too high for these people. Combining the sporting programs with broader physical activity programs, for instance, could both help in attracting inactive people and increasing their physical activity levels before engaging them into organized sports. For people who are inactive due to medical reasons, initial guidance by a physiotherapist could be helpful in decreasing fear of movement and physical limitations before participating in sport activities.

In general, it is important to know people’s reasons for inactivity and tailor HEPA promotion programs to the inactive target group [10]. This can be achieved by actively involving these people in the development of such programs using different formative research strategies (e.g. interviews, observations, focus groups) [36, 37]. The three sporting programs in the current study were pilot tested before advancing to broader implementation, with also inactive people participating in the pilot phase (4 to 14% of participants) [38]. However, it is not known to what extend their opinions were included in program design (as opposed to the opinions of insufficiently active people in general).

Finally, there might be some inactive people who cannot be persuaded to become physically active at all.

### Strengths and limitations of the study

This study was performed in the real-world sport setting, namely sports clubs, with participants voluntarily participating in the sporting programs. Therefore, results are directly transferable into practice. Furthermore, although data were collected in 2009, all three programs are still running (in the same way) in many different sports clubs in the Netherlands with on average 1.500 (Through 4 Days Marches) to 3.500 (Start to Run) participants each year. Thus, considering the recent interest for HEPA promotion in sports clubs, the findings are still relevant today (in 2018). These are strengths of this study. However, there are some limitations to this study which may have implications for interpretation of the results. First, it was not possible to determine why almost half of the participants dropped out of the study between the baseline and six-month measurement. Non-respondents were more likely to be female and somewhat younger, but there were no significant differences between respondents and non-respondents in baseline sport and physical activity behavior. The sporting programs themselves had a very low drop-out rate (i.e. between 2 and 3% of participants stopped with the programs). Furthermore, participants of this study were representative for the entire participant population of the individual sporting programs with regard to age and sex. Thus, it is unlikely that the study findings were influenced markedly by these losses to follow-up. Second, the use of self-report measures may have introduced social desirability biases, for instance, the over-reporting of physical activity. In this case, the percentage of active participants may be overestimated. Third, this study combined data of three sporting programs. This could be done, because the programs were very comparable with regard to their content, i.e. they used feasible sports, graded training programs and similar retention strategies. It is not known, however, to what extent these results are generalizable to other HEPA sporting programs, like programs that use less feasible sports (e.g. indoor sports for which special facilities or equipment are needed) or other training/retention strategies. Fourth, the baseline questionnaire contained a limited number of characteristics (individual, social) of participants. It is possible that other factors that were not measured in this questionnaire may also influence sport participation of insufficiently active people, such as factors in the physical (e.g. proximity to recreational facilities/sports club) or economic (e.g. costs for physical activity) environment [25, 27, 28]. Therefore, in future research, a larger number of factors should be taken into account. Finally, the number of insufficiently active participants was too low to perform more thorough analyses, for instance, to examine characteristics for different levels of baseline physical activity or to perform the analyses separately for males and females.

## Conclusions

Considering these limitations, this study does add to the knowledge base about who are the insufficiently active participants that benefit from HEPA promotion programs implemented in the sports club setting. The results suggest that HEPA sporting programs can be used to increase HEPA levels of insufficiently active people, especially of those who receive social support from family members with regard to their sport participation and already participate in a modest amount of moderate-intensity activity. The results may have implications for designing and implementing HEPA promotion programs in the sports clubs setting. For instance, it is important that promotional strategies and channels are tailored to the target group. Furthermore, strategies that promote family support may enhance the impact of the programs. Clearly further research is needed to understand the factors that influence sport and physical activity behavior of insufficiently active people and to develop effective strategies to improve HEPA-levels of this target group.

## Abbreviations

BMI: Body Mass Index
CI: Confidence interval
DNHPA: Dutch Norm for Health-enhancing Physical Activity
HEPA: Health-enhancing physical activity
MET: METabolic equivalent
MPA: Moderate-intensity physical activity
NAPSE: National Action Plan for Sport and Exercise
NSF: National Sports Federation
OR: Odds ratio
PA: Physical activity
SD: Standard deviation
SQUASH: Short QUestionnaire to ASsess Health-enhancing physical activity
TREND group: The Transparent Reporting of Evaluations with Nonrandomized Designs group
VPA: Vigorous-intensity physical activity
